# Changes in the Disparity of HIV Diagnosis Rates Among Black Women — United States, 2010–2014

**DOI:** 10.15585/mmwr.mm6604a3

**Published:** 2017-02-03

**Authors:** Donna Hubbard McCree, Madeline Sutton, Erin Bradley, Norma Harris

**Affiliations:** 1 Division of HIV/AIDS Prevention, CDC.

In 2015, black women represented 61% of human immunodeficiency virus (HIV) diagnoses among women (*1*). HIV diagnosis rates among women declined during 2010–2014 (*1*); however, whether the decline resulted in a decrease in the disparities between black women and Hispanic and white women was unknown. To assess whether a change in disparities occurred, CDC used three different measures of disparity: 1) the absolute rate difference (the difference between the group with the lowest rate and the group with the highest rate) (*2*); 2) the diagnosis disparity ratio* (the ratio of the difference between the group rate and the overall population rate to the overall rate); and 3) the Index of Disparity (the average of the differences between rates for specific groups and the total rate divided by the total rate, expressed as a percentage) (*3*). The absolute rate difference between black women and white women decreased annually, from 36.9 in 2010 to 28.3 in 2014. The diagnosis disparity ratio for black women decreased from 1.7 in 2010 to 1.2 in 2014. The Index of Disparity increased during 2010–2011, and then decreased each year during 2012–2014. Although disparities still exist, these findings indicate improvement. Expanding access to biomedical and behavioral interventions and research guided by social and structural determinants frameworks could close the remaining gap.

No standard test or broad consensus regarding the best single method for measuring and monitoring progress toward eliminating health disparities exists (*2*). Any assessment of a trend in health disparity needs to include both an absolute and a relative measure, and the assessment should use population-weighted measures to account for changes in the distribution of the population being monitored over time (*2*). In the absence of a statistical standard to measure HIV-related health disparities, CDC used three different measures to examine changes in the disparities of HIV diagnoses among black women during 2010–2014. The three measures were 1) the absolute rate difference, 2) the diagnosis disparity ratio, and 3) the Index of Disparity. The absolute rate difference and the diagnosis disparity ratio were selected because these measures are used by *Healthy People 2020*^†^ and the *National HIV/AIDS Strategy for the United States 2020* (NHAS),^§^ respectively, to measure progress in the social determinants of health and HIV diagnosis indicators for these initiatives. The Index of Disparity was selected because it represents a summary measure of disparity across population groups (*2*,*3*).

The absolute rate difference is a simple arithmetic difference that measures the absolute disparity between two groups for the same health status indicators (i.e., difference between the group with the lowest rate and the group with the highest rate) (*2*). *Healthy People 2020* uses a similar measure to monitor progress toward the social determinants health indicator (high school graduation rates by race/ethnicity). The diagnosis disparity ratio, the disparity measure for NHAS 2020 is similar to the absolute rate difference, but is a relative measure of disparity that assesses year-to-year progress toward an annual target (for NHAS 2020) (*4*). The overall HIV diagnosis rate was calculated by dividing the total number of HIV diagnoses by the U.S. Census population and multiplying the results by 100,000 (*4*); the HIV diagnosis rate for black women was calculated by dividing the number of HIV diagnoses in black women by the U.S. Census population for that group and multiplying the result by 100,000 (*4*). The ratio increases as the difference widens between a selected group and the overall population and decreases as the difference narrows (*4*). The diagnosis disparity ratios presented were obtained from the 2016 HIV surveillance supplemental report (*4*).

The Index of Disparity, also a relative measure of disparity, was determined by calculating the average difference of each group rate from the total rate, dividing that number by the total rate, and expressing the result as a percentage (*2*,*3*). The HIV diagnosis rates were obtained from the National Center for HIV/AIDS, Viral Hepatitis, STD, and TB Prevention Atlas.^¶^

During 2010–2014, rates of HIV diagnosis among black women ranged from 30.0 per 100,000 (2014) to 38.7 (2010) per 100,000 population. Rates were lower among Hispanic women, ranging from 6.2 (2012) to 7.8 (2010) and were lowest among white women, ranging from 1.6 (2012 and 2013) to 1.8 (2010) (Table).

**TABLE:** Rates of human immunodeficiency virus (HIV) diagnosis among adult women and adolescents aged ≥13 years, by race/ethnicity and disparity measure — United States, 2010–2014

Year	HIV diagnoses			Overall rate	Absolute rate difference^†^	Diagnosis disparity ratio^§^	Index of Disparity^¶^
	No. (rate)						
	Black	Hispanic	White				
2010	6,310 (38.7)	1,469 (7.8)	1,540 (1.8)	7.7	36.9	1.7	159.7
2011	5,856 (35.5)	1,351 (7.0)	1,506 (1.7)	6.9	33.8	1.5	164.5
2012	5,580 (33.4)	1,229 (6.2)	1,426 (1.6)	6.6	31.8	1.4	162.1
2013	5,227 (30.9)	1,279 (6.3)	1,418 (1.6)	6.3	29.3	1.3	155.6
2014	5,128 (30.0)	1,350 (6.5)	1,483 (1.7)	6.4	28.3	1.2	147.9

* Per 100,000 population.

^†^ Highest rate (black women) - lowest rate (white women).

^§^ Data report are taken from the following source: CDC. Monitoring selected national HIV prevention and care objectives by using HIV surveillance data—United States and 6 dependent areas, 2014. https://www.cdc.gov/hiv/library/reports/surveillance/.

^¶^ ([Average of individual group rates - Total rate]/Total rate) x 100.

The disparity in HIV diagnosis rates as measured by the absolute difference in rates between the group with the highest rate, black women, and the group with the lowest rate, white women, decreased annually, from 36.9 in 2010 to 28.3 in 2014 (Table). The diagnosis disparity ratio for black women also decreased annually, from 1.7 in 2010 to 1.2 in 2014 (*4*). The disparity in HIV diagnosis rates as measured by the Index of Disparity increased from 2010 to 2011, and then decreased each subsequent year from 2012 to 2014.

## Discussion

In 2015, black women were approximately 16 times more likely to receive a diagnosis of HIV infection than were white women, and they accounted for 61% of HIV diagnoses among women, compared with whites, who accounted for 19% of diagnoses, and Hispanics, who accounted for 15% (*1*). However, data indicate progress in reducing both HIV diagnoses and disparities in HIV diagnosis rates. Strategies available through public health systems and health care can maximize prevention measures targeting black women to decrease HIV diagnoses and reduce disparities. These strategies include routine HIV screening without cost sharing (*5*), recommendations for treatment of all persons living with HIV to prolong life and reduce transmission (*6*), and preexposure prophylaxis for women at increased risk for HIV infection (*7*). HIV testing is the entry point into medical care for persons living with HIV and is needed to initiate linkage to care and access to treatment that can prevent HIV transmission (*8*). During 2007–2010, CDC initiated the Expanded Testing Initiative (ETI) (*9*) that facilitated HIV screening and increased HIV diagnoses and linkage to care for disproportionately affected populations, particularly blacks. The ETI found that blacks accounted for 60% of tests and 70% of new HIV diagnoses (*9*). Based on available data, among all HIV infections newly diagnosed through ETI, 75.3% were successfully linked to HIV primary care (*9*). Because of the success of this initiative, the focused testing activities were integrated into the CDC flagship HIV Prevention Cooperative Agreement with Health Departments funded in 2012.

Further studies are needed to identify factors associated with decreases in these disparities and to investigate whether the decreases are uniform or differ systematically (e.g., by geographical location) across the United States, and accelerate progress toward decreasing HIV infection and disparities among women. Future research needs to include a focus on access to testing and treatment for black women and men (the majority of women acquire HIV infection through sexual contact with men known to have or be at high risk for HIV infection) and social determinants of health, including poverty and suboptimal educational and employment opportunities that disproportionately affect some black communities and might impede HIV prevention programs. Theoretical frameworks that account for the interplay between interpersonal, social, and structural factors (*10*) might contribute to understanding the role of social determinants in reducing disparities.

The findings in this report are subject to at least two limitations. First, the measures used were selected based on their use in national indicators of progress toward reducing disparities and to provide summary measures of disparity across racial/ethnic groups of women. Use of other measures of disparity can be found in the scientific literature, and use of these other measures might have yielded different results. Second, the diagnosis estimates in this report are based on national surveillance data and are likely affected by some underreporting and reporting delays.

Commonly used measures of disparity indicate decreases in disparity of HIV diagnosis rates among black women compared with Hispanic and white women. However, disparities persist, and eliminating these HIV-related health disparities remains a national goal. Maintaining momentum in advances toward health equity could potentially include expanding access to biomedical and behavioral interventions and targeted, culturally sensitive research guided by social and structural determinants frameworks.

**Summary**

**What is already known about this topic?**

Human immunodeficiency virus (HIV) infection rates among women declined 40% between 2005 and 2014 with the largest decline, 42%, occurring in black women. Black women represented 59% of women living with HIV infection at the end of 2014 and 61% of HIV diagnoses among women in 2015.

**What is added by this report?**

The disparity in HIV diagnosis rates among black women compared with rates among Hispanic and white women, as calculated by three different measures (the absolute rate difference, the diagnosis disparity ratio and the Index of Disparity), decreased in 2014 compared with 2010.

**What are the implications for public health practice?**

This decrease in all three measures of disparity suggests that prevention measures targeting women might be reducing HIV infections in black women. Because black women remain disproportionately affected by HIV infection, additional interventions that are culturally tailored to them might aid in further reducing the prevalence of HIV among this group.

## Footnotes

* National HIV/AIDS Strategy for the United States: Updated to 2020 Indicator Supplement. https://www.aids.gov/federal-resources/national-hiv-aids-strategy/nhas-2020-indicators.pdf.

† Healthy People 2020 Leading Health Indicators: Social Determinants of Health 2014. https://www.healthypeople.gov/sites/default/files/HP2020_LHI_Soc_Determ_0.pdf.

§ National HIV/AIDS Strategy for the United States: Updated to 2020. https://www.aids.gov/federal-resources/national-hiv-aids-strategy/nhas-update.pdf.

¶ https://www.cdc.gov/nchhstp/atlas/.
